# The effects of canagliflozin compared to sitagliptin on cardiorespiratory fitness in type 2 diabetes mellitus and heart failure with reduced ejection fraction: The CANA‐HF study

**DOI:** 10.1002/dmrr.3335

**Published:** 2020-06-15

**Authors:** Salvatore Carbone, Hayley E. Billingsley, Justin M. Canada, Edoardo Bressi, Brando Rotelli, Dinesh Kadariya, Dave L. Dixon, Roshanak Markley, Cory R. Trankle, Richard Cooke, Krishnasree Rao, Keyur B. Shah, Horacio Medina de Chazal, Juan Guido Chiabrando, Alessandra Vecchié, Megan Dell, Virginia L. Mihalick, Roberta Bogaev, Linda Hart, Benjamin W. Van Tassell, Ross Arena, Francesco S. Celi, Antonio Abbate

**Affiliations:** ^1^ Department of Kinesiology & Health Sciences, College of Humanities & Sciences Virginia Commonwealth University Richmond Virginia USA; ^2^ Division of Cardiology, Department of Internal Medicine, VCU Pauley Heart Center Virginia Commonwealth University Richmond Virginia USA; ^3^ Department of Pharmacotherapy and & Outcomes Science, School of Pharmacy Virginia Commonwealth University Richmond Virginia USA; ^4^ Advanced Heart Failure Center Bon Secours Heart & Vascular Institute Richmond Virginia USA; ^5^ Department of Physical Therapy, College of Applied Health Sciences University of Illinois at Chicago Chicago Illinois USA; ^6^ TotalCardiology Research Network Calgary Alberta Canada; ^7^ Division of Endocrinology Diabetes and Metabolism, Department of Internal Medicine Virginia Commonwealth University Richmond Virginia USA

**Keywords:** cardiorespiratory fitness, diabetes mellitus, DPP4 inhibitors, heart failure, SGLT2 inhibitors

## Abstract

**Background:**

Canagliflozin reduces hospitalizations for heart failure (HF) in type 2 diabetes mellitus (T2DM). Its effect on cardiorespiratory fitness and cardiac function in patients with established HF with reduced ejection fraction (HFrEF) is unknown.

**Methods:**

We conducted a double‐blind randomized controlled trial of canagliflozin 100 mg or sitagliptin 100 mg daily for 12 weeks in 88 patients, and measured peak oxygen consumption (VO_2_) and minute ventilation/carbon dioxide production (VE/VCO_2_) slope (co‐primary endpoints for repeated measure ANOVA time_x_group interaction), lean peak VO_2_, ventilatory anaerobic threshold (VAT), cardiac function and quality of life (ie, Minnesota Living with Heart Failure Questionnaire [MLHFQ]), at baseline and 12‐week follow‐up.

**Results:**

The study was terminated early due to the new guidelines recommending canagliflozin over sitagliptin in HF: 17 patients were assigned to canagliflozin and 19 to sitagliptin, total of 36 patients. There were no significant changes in peak VO_2_ and VE/VCO_2_ slope between the two groups (*P* = .083 and *P* = .98, respectively). Canagliflozin improved lean peak VO_2_ (+2.4 mL kg_LM_^−1^ min^−1^, *P* = .036), VAT (+1.5 mL kg^−1^ min^−1^, *P* = .012) and VO_2_ matched for respiratory exchange ratio (+2.4 mL Kg^−1^ min^−1^, *P* = .002) compared to sitagliptin. Canagliflozin also reduced MLHFQ score (−12.1, *P* = .018).

**Conclusions:**

In this small and short‐term study of patients with T2DM and HFrEF, interrupted early after only 36 patients, canagliflozin did not improve the primary endpoints of peak VO_2_ or VE/VCO_2_ slope compared to sitagliptin, while showing favourable trends observed on several additional surrogate endpoints such as lean peak VO_2_, VAT and quality of life.

## 1 INTRODUCTION

Heart failure (HF) remains one of the most common comorbidities in type 2 diabetes mellitus (T2DM).^1^ The sodium‐glucose co‐transporter (SGLT)‐2 inhibitors, canagliflozin,^2^ dapagliflozin^3^ and empagliflozin,^4^ are the only glucose‐lowering agents that reduce the incidence of HF and HF‐related hospitalizations in patients with T2DM, and dapagliflozin also in patients without T2DM and established HF with reduced ejection fraction (HFrEF).^5^ The mechanisms explaining these benefits appear, at least in part, independent of glycemic control.

The cardiovascular outcomes trials with the SGLT2 inhibitors canagliflozin and empagliflozin do not provide data on the forms of HF and HF‐related events being prevented, with a small percentage of patients having HF at baseline. Dapagliflozin reduced the risk of worsening HF or death from cardiovascular causes in established HFrEF patients and improved quality of life (QoL) in patients with and without T2DM.^5^

Exercise intolerance is a hallmark consequence of HF, typically defined as a reduction in cardiorespiratory fitness (CRF) measured as a reduced peak oxygen consumption (VO_2_) during cardiopulmonary exercise testing (CPX) and largely responsible for the reduced QoL in HF.^6^ Peak VO_2_ is a strong prognostic factor in HFrEF, and its improvements have been associated with long‐term reduction of clinical events, making it a strong surrogate outcome measure.^7^ Despite several therapeutics able to improve CRF,^6^, ^8^ patients with HFrEF still present with a markedly reduced CRF. The effects of SGLT2 inhibitors on CRF in patients with established HF have been only minimally investigated,^9^, ^10^ and, to date, no randomized controlled trials comparing the effects of SGLT2 inhibitors on CRF with other glucose‐lowering agents have been performed.

We investigated whether canagliflozin improved peak VO_2_ and ventilatory efficiency in patients with T2DM and HFrEF compared to sitagliptin, a dipeptidyl peptidase (DPP)4 inhibitor with proven cardiovascular safety with glucose‐lowering efficacy similar to SGLT2 inhibitors.^11^ We hypothesized that improvements in CRF would be independent of glycemic control.

## 2 METHODS

### 2.1 Study design

**FIGURE 1:**
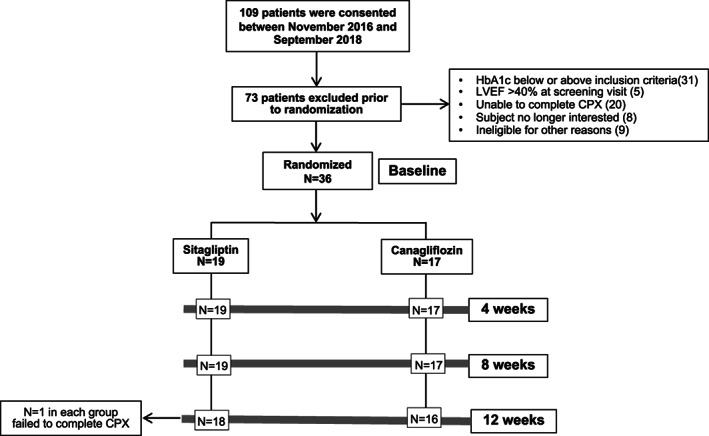
Screening and enrollment. CPX, cardiopulmonary exercise testing; HbA1c, glycated haemoglobin; LVEF, left ventricular ejection fraction

The CANA‐HF study was a double blind, randomized, controlled study investigating the effects of canagliflozin 100 mg daily compared to sitagliptin 100 mg daily for 12 weeks in 88 patients with T2DM and stable chronic HFrEF on CRF started on September 2016. On 22 October 2018, due to the new guidelines released by the American Diabetes Association^12^ and the European Association for the Study of Diabetes^13^ recommending the use of SGLT2 inhibitors over sitagliptin in patients with HF, the study was interrupted prematurely after consultation with the sponsor, as we believed that an equipoise no longer existed between the two treatments. Thus, we enrolled a total of 36 patients (Figure 1).

### 2.2 Inclusion and exclusion criteria

Briefly, stable patients with symptomatic HF (New York Heart Association [NYHA] class II‐III) with a left ventricular ejection fraction (LVEF) ≤40%, T2DM and reduced CRF were enrolled (Supporting Information for full list of inclusion and exclusion criteria). The Western Institutional Review Board approved the study and all patients provided written informed consent.

### 2.3 Cardiopulmonary exercise testing

At baseline and 12 weeks subjects underwent a symptom‐limited CPX, using a metabolic cart interfaced with a treadmill utilizing a conservative ramping protocol wherein the speed and grade increased approximately 0.6 estimated metabolic equivalents every minute, as previously described.^14^ Peak VO_2_ was defined as the highest 10‐second interval average during the last 30 seconds of exercise.^6^ The VE/VCO_2_ slope was calculated using 10‐second averaged VE and VCO_2_ data from the initiation of exercise to peak that was inserted into spreadsheet software (Microsoft Excel, Microsoft Corp., Bellevue, Washington) to calculate the slope via least squares linear regression.^6^ The VE/VCO_2_ slope is a marker of HF severity defined as minute ventilation/carbon dioxide production (VE/VCO_2_) slope.

### 2.4 Doppler echocardiography

At baseline and at 12 weeks we performed resting transthoracic Doppler echocardiography.^15^ We measured LV end‐diastolic and LV end‐systolic volumes, LVEF, and early transmitral velocity (E) on pulsed‐wave Doppler spectra and early mitral annular velocities by tissue Doppler averaged between the lateral and septal (E′) annulus; these measures were used to calculate the E/E′ ratio. We also measured deceleration time (DT) and calculated the E′ velocity indexed by DT (E′_DT_).^16^

### 2.5 Quality of life

We measured QoL at baseline and 12 weeks using the Minnesota Living with Heart Failure Questionnaire (MLHFQ). The MLHFQ is a 21‐item graded questionnaire used to assess the impairments in QoL in HF, higher scores reflect a greater HF symptom burden.^17^

### 2.6 Anthropometrics, fluid status and blood pressure

We measured body weight and height to calculate body mass index (BMI) and waist circumference at baseline and at 12 weeks.^18^ We assessed lean mass (LM) to calculate lean peak VO_2_ using dual‐energy X‐Ray Absorptiometry (DEXA) (QDR 4500a, Hologic, Marlborough, Massachusetts)^19^ and changes in fluid status: total body water (TBW), intracellular water (ICW) and extracellular water (ECW) using bioelectrical impedance analysis (BIA; Quantum IV, RJL System, Clinton Township, Michigan) at baseline and 12 weeks. Resting blood pressure was measured at baseline and after 12 weeks using a Tango automated blood pressure system (Suntech Medical, Morrisville, North Carolina).

### 2.7 Biomarkers

We measured non‐fasting biomarkers: HbA1c, N‐terminal pro‐brain natriuretic peptide (NTproBNP) and glomerular filtration rate (GFR), at baseline and 12 weeks.

### 2.8 Dietary intake

Because canagliflozin increases glucose and sodium urinary excretion resulting in a modest daily caloric deficit, we also assessed dietary caloric and sodium intake: the research dietitian performed a standardized 5‐pass 24‐hour dietary recall.^20^ Food consumption was converted in calories and sodium using the Nutrition Data System for Research 2016.^20^

### 2.9 Study endpoints

The co‐primary endpoints for the study were changes from baseline in peak VO_2_ and the VE/VCO_2_ slope after 12 weeks of treatment with canagliflozin 100 mg daily or sitagliptin 100 mg daily. We measured QoL using the MLHFQ and additional CRF variable: ventilatory anaerobic threshold (VAT), and DEXA‐measured lean peak VO_2_.^6^, ^21^ Lean peak VO_2_ has been proposed to be superior to peak VO_2_ in determining prognosis in patients with HFrEF, especially in patients with obesity, in which peak VO_2_ may underestimate the overall level of CRF.^6^, ^21^ We performed a post‐hoc exploratory analysis to investigate the effects of canagliflozin vs sitagliptin on respiratory exchange ratio (RER)‐matched VO_2_ after 12 weeks, using the VO_2_ values corresponding to the lowest RER achieved between baseline and follow‐up. Such analysis allowed for correction of differences in RER between baseline and follow‐up. Additional exploratory analyses included measures of resting cardiac function, anthropometrics, body water and blood pressure.

### 2.10 Statistical analysis and sample size calculation

Statistical analyses were performed with SPSS 24.0 package (Chicago, Illinois). Continuous data are reported as means and standard deviations for normally distributed variables or median and interquartile range for potential deviation from the Gaussian distribution, and discrete variables were reported as N and %. We used the nonparametric Wilcoxon signed‐rank test for repeated measures to assess the within‐group related changes compared to baseline. The differences in interval changes between the two groups were compared using a random‐effect analysis of variance model for repeated measures to analyse the effects of time and group allocation. Unadjusted *P*‐values were reported throughout, with statistical significance set at the two‐tailed .025 level for the co‐primary endpoints and .05 for the additional analyses. Missing data were omitted and not imputed and the cases excluded from the related analysis. A sample size of 40 patients per group provided sufficient power to detect a MD in the interval change in peak VO_2_ of 1.50 ± 1.76 mL kg^−1^ min^−1^ expected with canagliflozin compared to sitagliptin, which we predicted to have no significant effects on peak VO_2_ (0 ± 1.76 mL kg^−1^ min^−1^; power of >90%, *α* value .025 two‐sided).^9^, ^22^, ^23^ We initially anticipated that a sample size of 88 patients would allow for a 10% loss to follow‐up. Study enrolment was, however, interrupted early, ultimately enrolling 36 individuals, providing a sufficient statistical power to detect a MD in the interval change in peak VO_2_ of 1.50 ± 1.76 mL kg^−1^ min^−1^ with canagliflozin compared to sitagliptin (0 ± 1.76 mL kg^−1^ min^−1^; power of 70%, *α* value .025 two‐sided).

## 3 RESULTS

### 3.1 Baseline characteristics

No statistically significant differences were found between the two groups at baseline, except for peak RER, which was significantly lower in the sitagliptin group compared to canagliflozin (Table 1). Patients randomized to canagliflozin tended to be more likely to receive insulin, had a lower BMI, and lower NTproBNP, although the differences did not reach statistical significance. Patients were followed for a mean follow‐up of 93 days.

**TABLE 1:** Baseline demographics and clinical characteristics

	Sitagliptin n = 19	Canaglifozin n = 17	*P* value
Male	15 (78.9)	13 (76.5)	.86
Age, y	54.3 ± 8.8	58 ± 6.1	.15
Caucasian	7 (36.8)	10 (58.8)	.19
Non‐Hispanic	18 (94.7)	14 (82.4)	.33
Body mass index, kg/m^2^	38.8 ± 7	34.5 ± 6.6	.06
Systolic blood pressure, mmHg	130.4 ± 20.4	130.8 ± 17.1	.96
Diastolic blood pressure, mmHg	72 ± 13.4	72.5 ± 13.2	.93
Hypertension	17 (89.5)	16 (94.1)	.62
Hyperlipidemia	16 (84.2)	15 (88.2)	.73
Atrial fibrillation	5 (26.3)	4 (23.5)	1
Current tobacco	3 (15.8)	5 (29.4)	.43
Coronary artery disease	7 (36.8)	9 (52.9)	.33
Myocardial infarction	5 (26.3)	4 (23.5)	1
CABG	0	3 (17.6)	.10
COPD	2 (10.5)	3 (17.6)	.65
Peripheral artery disease	3 (15.8)	3 (17.6)	1
Stroke/TIA	3 (15.8)	1 (5.9)	.61
*Heart failure therapy*			
Aldosterone blockers	13 (68.4)	9 (52.9)	.34
Angiotensin blockers	11 (57.9)	14 (82.4)	.11
ARNI	4 (21.1)	2 (11.8)	.66
Aspirin	12 (63.2)	13 (76.5)	.39
Digoxin	0	1 (5.9)	.47
DOACs	5 (26.3)	3 (17.6)	.70
Hydralazine	3 (15.8)	3 (17.6)	1
ICD	7 (36.8)	5 (29.4)	.64
Loop diuretics	18 (94.7)	13 (76.5)	.11
Nitrates	6 (31.6)	3 (17.6)	.45
Statins	17 (89.5)	17 (100)	.17
Warfarin	3 (15.8)	1 (5.9)	.61
β‐adrenergic receptor blockers	18 (94.7)	16 (94.1)	.94
*Glucose‐lowering agents*			
Biguanides	10 (52.6)	10 (58.8)	.71
DPP4 inhibitors	1 (5.3)	0	.53
GLP1 receptor agonists	0	1 (5.9)	.47
Insulin	6 (31.6)	11 (64.7)	.051
Sulfonylureas	5 (26.3)	1 (5.9)	.13
*NYHA class and quality of life*			
II	12 (63.2)	10 (58.8)	.93
III	7 (36.8)	7 (41.2)	.79
MLHFQ score	38.4 ± 26.6	49.2 ± 26.8	.43
*Doppler echocardiography parameters*			
LVEF, %	27.0 ± 6.8	31.6 ± 7.5	.28
E/e′ ratio	13.8 (11.4‐18.2)	12.4 ± (8.6‐30.0)	.42
Deceleration time velocity, ms	179 (153‐211)	205 (159‐263)	.57
LVESV index	61 (43‐76)	36 (27‐57)	.42
LVEDV index	78 (65‐108)	58 (42‐83)	.42
*Cardiopulmonary exercise testing parameters*			
Exercise time, s	551 ± 108	561 ± 143	.82
Peak VO_2_, mL Kg^−1^ min^−1^	15.3 ± 3.5	16.2 ± 3.4	.43
Peak VO_2_, mL Kg_LM_^−1^ min^−1^	22.9 ± 5.0	25.2 ± 5.4	.27
Respiratory exchange ratio	1.04 ± 0.4	1.12 ± 0.1	**.01**
VAT, mL Kg^−1^ min^−1^	11.6 ± 3	11.7 ± 2.2	.95
VE/VCO_2_ slope	32.6 ± 7.2	34 ± 6.1	.52
*Laboratory*			
Creatinine (mg/dL)	1.09 ± 0.32	1.12 ± 0.24	.74
Glomerular filtration rate (mL min^−1^/1.73 m^2^)	83.3 ± 22	74.7 ± 19	.22
HbA1c (%)	8.3 ± 1.3	8.3 ± 1.4	.91
Haemoglobin (g/dL)	13.1 ± 1.6	13.6 ± 1.6	.37
NTproBNP (pg/mL)	492 (330‐961)	243 (74‐750)	.10
Red blood cells (×10^6^/mL)	4.5 ± 0.6	4.6 ± 0.6	.66
White blood cells (×10^3^/mL)	7.2 ± 2.2	7.6 ± 2.3	.62

Abbreviations: CABG, coronary artery bypass surgery; COPD, chronic obstructive pulmonary disease; CRT, cardiac resynchronization therapy; DOACs, direct oral anti‐coagulants; DPP, dipeptidyl peptidase; GLP, glucagon‐like peptide; HbA1c, glycosylated haemoglobin; ICD, implantable cardiac defibrillator; LM, lean mass; LVEDV, left ventricular end‐diastolic volume; LVEF, left ventricular ejection fraction; LVESV, left ventricular end‐systolic volume; MLHFQ, Minnesota Living with Heart Failure Questionnaire; NTproBNP, N‐terminal pro b‐type natriuretic peptide; NYHA, New York Heart association; TIA, transient ischemic attack; VAT, ventilatory anaerobic threshold; VE/VCO_2_, minute ventilation/carbon dioxide production; VO_2_, oxygen consumption.

### 3.2 Cardiorespiratory fitness

**FIGURE 2:**
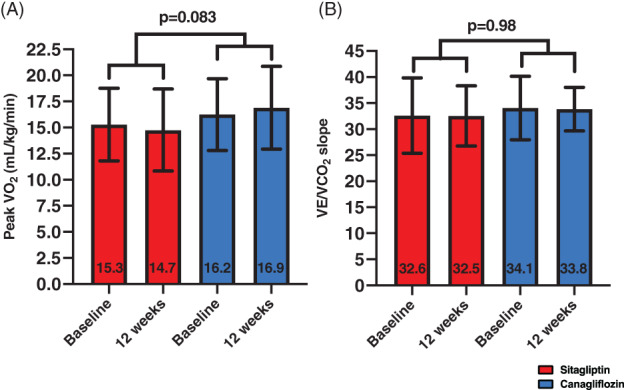
Effects of treatments on peak oxygen consumption (VO_2_) and minute ventilation/carbon dioxide production slope (VE/VCO_2_ slope). Treatment with canagliflozin nor sitagliptin did not result in significant changes in peak VO_2_, A and VE/VCO_2_ slope, B. Data are presented as mean ± SD

We did not observe any statistically significant improvements in the co‐primary endpoints of the study in any of the groups (Table 2). We did not detect any significant within‐group differences in the canagliflozin (peak VO_2_: from 16.2 ± 3.4 to 16.9 ± 4.0 mL kg^−1^ min^−1^, *P* = .23; VE/VCO_2_ slope: from 34.1 ± 6.1 to 33.8 ± 4.2, *P* = .66) and sitagliptin groups (peak VO_2_ from 15.3 ± 3.5 to 14.7 ± 3.9 mL kg^−1^ min^−1^, *P* = .16; VE/VCO_2_ slope: from 32.6 ± 7.2 to 32.5 ± 5.8, *P* = .65), nor between‐group differences (peak VO_2_ mean difference [MD] between groups: +1.3 mL kg^−1^ min^−1^, *P* = .083; VE/VCO_2_: MD between groups: −0.2, *P* = .98; Figure 2).

**TABLE 2:** Cardiorespiratory fitness and cardiac function

Cardiorespiratory fitness variables	Sitagliptin n = 19			Canaglifozin n = 17			*P* value (time_X_group interaction)
	Baseline	12 wk	*P* value	Baseline	12 wk	*P* value	
Peak VO_2_, mL min^−1^	1769 ± 489	1710 ± 392	0.21	1583 ± 409	1600 ± 454	0.86	0.15
VE/VCO_2_ slope	32.6 ± 7.2	32.5 ± 5.8	0.65	34 ± 6.1	33.8 ± 4.2	0.66	0.98
Peak VO_2_, mL Kg^−1^ min^−1^	15.3 ± 3.5	14.8 ± 3.9	0.16	16.2 ± 3.4	16.9 ± 4.0	0.23	0.08
Peak VO_2_, mL Kg_LM_^−1^ min^−1^	22.9 ± 5.0	22.0 ± 5.4	0.16	25.2 ± 5.0	26.7 ± 5.3	0.16	**0.036**
Peak VO_2_ pulse, mL min^−1^	13.9 ± 3.5	13.6 ± 3.6	0.78	12.7 ± 3.1	12.9 ± 3.5	0.53	0.46
VAT, mL Kg^−1^ min^−1^	11.6 ± 3.0	11.8 ± 2.9	0.93	11.7 ± 2.2	13.1 ± 2.3	0.004	**0.01**
OUES	2.11 ± 0.58	2.06 ± 0.55	0.64	1.72 ± 0.36	1.80 ± 0.43	0.09	0.15
RER‐matched VO_2_, mL Kg^−1^ min^−1^	14.4 ± 3.9	14.7 ± 3.9	0.26	14.2 ± 4.2	16.9 ± 3.9	0.001	**0.002**
Respiratory exchange ratio	1.04 ± 0.04	1.03 ± 0.07	0.38	1.12 ± 0.11	1.0 ± 0.07	<0.001	**0.001**
Exercise time, s	551 ± 108	534 ± 120	0.12	561 ± 143	573 ± 151	0.69	0.13
Resting heart rate, bpm	71 ± 10	73 ± 10	0.89	70 ± 10	69 ± 11	0.23	0.43
Peak heart rate, bpm	129 ± 23	126 ± 24	0.18	125 ± 17	125 ± 15	0.82	0.46
Resting systolic blood pressure, mmHg	130 ± 20	124 ± 15	0.27	131. ±17	130 ± 17	0.91	0.37
Peak systolic blood pressure, mmHg	159 ± 29	162 ± 26	0.42	174 ± 31	174 ± 30	0.26	0.19
Resting diastolic blood pressure, mmHg	71 ± 16	69 ± 13	0.39	72 ± 13	74 ± 14	0.78	0.99
Peak diastolic blood pressure, mmHg	72 ± 13	74 ± 13	0.41	77 ± 14	76 ± 16	0.85	0.25

*Echocardiographic Variables*	*Sitagliptin n = 19*			*Canaglifozin n = 17*			*P value (time_X_group interaction)*
	*Baseline*	*12 wk*	*P value*	*Baseline*	*12 wk*	*P value*	
Deceleration time velocity, ms	179 (153–211)	187 (159–216)	0.13	205 (159–263)	193 (152–233)	0.08	**0.02**
E/E′	13.9 ± 4.6	13.4 ± 4.6	0.45	18.5 ± 13.2	16.7 ± 9.4	0.43	0.63
E/DT	0.03 ± 0.01	0.04 ± 0.02	0.82	0.03 ± 0.01	0.03 ± 0.01	0.06	0.46
LVESV index	61 (43–76)	51 (44‐61)	0.01	36 (27‐57)	37 (23‐47)	0.02	0.92
LVEDV index	78 (65–108)	81 (58‐89)	0.27	58 (42–83)	54 (42‐73)	0.86	0.68
LVEF, %	27.0 ± 6.8	30.3 ± 10.4	0.18	31.6 ± 7.5	38.8 ± 10.9	0.006	0.21
TAPSE, cm	2.18 ± 0.47	2.04 ± 0.46	0.34	2.15 ± 0.42	2.19 ± 0.54	0.29	0.16

Abbreviations: DT, deceleration time; LVEDV, left ventricular end‐diastolic volume; LVEF, left ventricular ejection fraction; LVESV, left ventricular end‐systolic volume; OUES, oxygen uptake efficiency slope; RER, respiratory exchange ratio; TAPSE, tricuspid annular plane systolic excursion; VAT, ventilatory anaerobic threshold; VE/VCO_2_, minute ventilation/carbon dioxide production; VO_2_, oxygen consumption.

**FIGURE 3:**
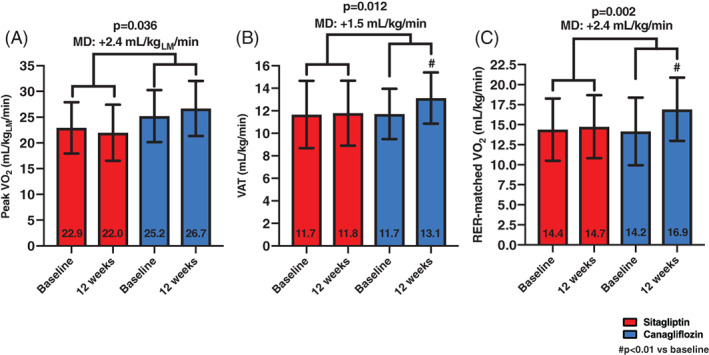
Effects of treatments on exploratory cardiopulmonary exercise testing (CPX) variables. Canagliflozin resulted in significant improvements in lean peak oxygen consumption (VO_2_), A, ventilatory anaerobic threshold (VAT), B, and RER‐matched VO_2_, C, compared to sitagliptin in a time_x_group interaction. Data are presented as mean ± SD. LM, lean mass measured with dual‐energy X‐ray absorptiometry; MD, mean difference between groups

Canagliflozin improved lean peak VO_2_ compared to sitagliptin (mean difference [MD] between changes of +2.4 mL kg_LM_^−1^ min^−1^, *P* = .036; canagliflozin within group: from 25.2 to 26.7 mL kg_LM_^−1^ min^−1^, *P* = .15; sitagliptin within group: from 22.9 to 22.0 mL kg_LM_^−1^ min^−1^, *P* = .15; Figure 3). Canagliflozin also induced a significant improvement in VAT compared to sitagliptin (MD between groups: +1.5 mL kg^−1^ min^−1^, *P* = .012; canagliflozin within group: from 11.7 ± 2.2 to 13.1 ± 2.3 mL kg^−1^ min^−1^, *P* = .004; sitagliptin within group: from 11.7 ± 3.0 to 11.8 ± 2.9 mL kg^−1^ min^−1^, *P* = .93; Figure 3).

RER was significantly reduced at 12 weeks in the canagliflozin group (MD between groups: −0.10, *P* < .001; from 1.12 ± 0.11 to 1.00 ± 0.08, in the canagliflozin group, *P* < .001, and from 1.04 ± 0.04 to 1.02 ± 0.07, in the sitagliptin group, *P* = .38). Therefore, we calculated the RER‐matched VO_2_, allowing for a VO_2_ comparison at similar levels of exercise effort. Canagliflozin showed a significant improvement in RER‐matched VO_2_ (MD between groups: +2.4 mL kg^−1^ min^−1^, *P* = .002; from 14.2 ± 4.2 to 16.9 ± 3.9 mL kg^−1^ min^−1^, in canagliflozin, *P* = .001; and from 15.3 ± 3.5 to 14.7 ± 3.9 mL kg^−1^ min^−1^, with sitagliptin, *P* = .26; Figure 3).

### 3.3 Quality of life

**FIGURE 4:**
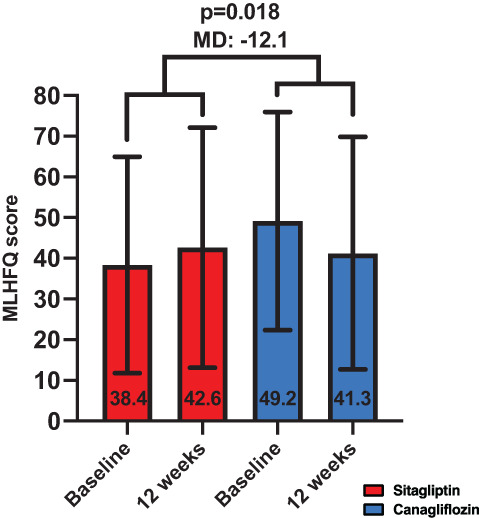
Effects of treatments on quality of life. Canagliflozin improved quality of life (QoL) as shown in the Minnesota Living with Heart Failure Questionnaire (MLHFQ) score, where a reduction in the score suggests improvements in QoL. Data are presented as mean ± SD. MD, mean difference between groups

Canagliflozin reduced the MLHFQ score compared to sitagliptin, where a reduction of the score reflects improvements in QoL (MD between groups: −12.1, *P* = .018; canagliflozin within group: from 49.2 ± 26.8 to 41.3 ± 28.6, *P* = .073; sitagliptin from 38.4 ± 26.6 to 42.6 ± 29.5, *P* = .14; Figure 4).

### 3.4 Doppler echocardiography

**FIGURE 5:**
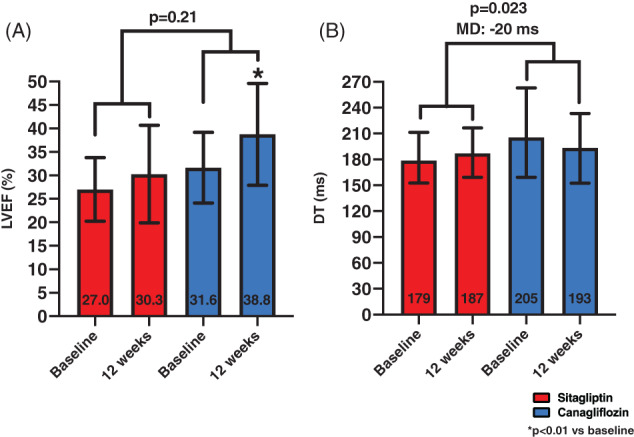
Effects of treatments on cardiac function. Canagliflozin was associated with improved systolic and diastolic function compared to baseline. Specifically, we observed improvements in left ventricular ejection fraction (LVEF), A, and deceleration time (DT), B. When compared to sitagliptin at time_x_group interaction analysis, however, the difference in LVEF was no longer statistically significant, while the difference with DT remained statistically significant. Data are presented as mean ± SD for LVEF, and median and interquartile ranges for DT. MD, mean difference between groups

Canagliflozin was associated with a small reduction in DT compared with sitagliptin (MD between groups: −20 ms, *P* = .023; canagliflozin: from 205 [159‐263] to 193 [152‐233] ms, *P* = .078; sitagliptin: from 179 [153‐211] to 187 [159‐216] ms, *P* = .13; Table 2 and Figure 5). No changes in E/e′ were observed in the within group as well as time_x_group interaction (Table 2). Treatment with canagliflozin, but not sitagliptin was associated with a significant within‐group improvement in LVEF (canagliflozin: from 31.6 ± 7.5 to 38.8 ± 10.9%, *P* = .006; sitagliptin: from 27.0 ± 6.8 to 30.3 ± 10.4%, *P* = .18), however, there was no significant time_x_group interaction (MD between groups: +3.9%, *P* = .21; Table 2 and Figure 5).

### 3.5 Biomarkers

Glycemic control (ie, HbA1c) did not differ between canagliflozin and sitagliptin (*P* = .63 for time_x_group interaction; canagliflozin: from 8.3 ± 1.4 to 8.0 ± 1.0%, *P* = .29; sitagliptin: from 8.3 ± 1.3 to 7.6% ± 1.4, *P* = .10;). No significant changes for NTproBNP were found at time_x_group interaction (*P* = .47; canagliflozin within group: from 243 [74‐750] to 257 [106‐481] pg/mL, *P* = .83; sitagliptin: from 492 [330‐961] to 512 [132‐895] pg/mL, *P* = .91). No significant changes in estimated GFR were observed at time_x_group interaction analysis (MD between groups: +8.9 mL/min/1.73 m^2^, *P* = .09; canagliflozin: from 74.7 ± 18.9 to 79.2 ± 19.8 mL/min/1.73 m^2^, *P* = .20; sitagliptin: from 83.3 ± 22.1 to 78.9 ± 24.2 mL/min/1.73 m^2^, *P* = .60).

### 3.6 Dietary Intake

We did not observe any significant changes in reported daily caloric intake (time_x_group interaction analysis, *P* = .15) (canagliflozin: from 1635 ± 645 to 1852 ± 784 kcal, *P* = .21; sitagliptin: from 1846 ± 692 to 1702 ± 495 kcal, *P* = .53). Sodium intake was not significantly affected by either intervention at time_x_group interaction (*P* = .73; canagliflozin: from 3241 ± 1250 to 3470 ± 1697 mg, *P* = .86; sitagliptin: from 3145 ± 987 to 3183 ± 1253 mg, *P* = .84).

### 3.7 Anthropometrics, fluid status and blood pressure

Neither canagliflozin nor sitagliptin significantly affected blood pressure or fluid status (all *P* > .05; Figure S1). We observed a non‐significant trend for BMI reduction favouring canagliflozin (canagliflozin: from 34.5 ± 6.6 to 34.1 ± 6.7 kg/m^2^, *P* = .084; sitagliptin: from 38.9 ± 7.0 to 38.8 ± 7.1 kg/m^2^, *P* = .58; *P* = .053 for time_x_group interaction analysis). We did not observe a significant difference in waist circumference between groups (canagliflozin: from 116.7 ± 15.2 to 114.3 ± 15.3 cm, *P* = .073; sitagliptin: from 126.3 ± 17.0 to 124.4 ± 15.0 cm, *P* = .57; *P* = .14 for time_x_group interaction analysis).

### 3.8 Adverse events

As expected, the overall number of adverse events was low (Table 3). One event of worsening HF with sitagliptin was reported. No events of amputations, diabetic ketoacidosis nor fractures were reported.

**TABLE 3:** Adverse events

	Sitagliptin n = 19	Canaglifozin n = 17
Acute kidney injury	3 (15.8)	4 (23.5)
Arrhythmic events	1 (5.3)	1 (5.9)
Genital infections	1 (5.3)	1 (5.9)
Hypoglycemia	0	2 (11.8)
Hypotensive events	1 (5.3)	2 (11.8)
Rehospitalizations for any cause	1 (5.3)	1 (5.9)
Unplanned revascularizations	0	1 (5.9)
Worsening of heart failure	1 (5.3)	0

## 4 DISCUSSION

In the current study, we have shown that 12‐week treatment with an SGLT2 inhibitor, canagliflozin, was well‐tolerated and did not improve the primary endpoints of peak VO_2_ and VE/VCO_2_ slope. Canagliflozin, however, improved several surrogate functional markers of CRF, cardiac function and QoL compared to a DPP4 inhibitor, sitagliptin, in patients with stable HFrEF and T2DM. The trial was prematurely halted after only 40% of subjects were enrolled because of concerns given recent guideline recommendations to preferentially use SGLT2 inhibitors, instead of DPP4 inhibitors, in patients with T2DM and HF, thus, significantly reducing the overall power of the study. Possibly due to an early termination after 36 subjects, the study failed to meet the co‐primary endpoints, showing, however, a MD of 1.3 mL kg^−1^ min^−1^ for peak VO_2_ that failed to reach the pre‐specified statistical significance (*P* = .083).

Canagliflozin was associated with a significant improvement in QoL and additional CPX variables that have been found to consistently predict prognosis in patients with HF, and have proven to be more sensitive than peak VO_2_, such as lean peak VO_2_ and VAT.^6^, ^21^ Treatment with canagliflozin was associated with a significant reduction in the MLHFQ, indicating improved QoL related to improvements in HF‐related symptoms.

In patients with HF, lean peak VO_2_ may be a stronger prognosticator than peak VO_2_, particularly in individuals with obesity, in which peak VO_2_ adjusted by total body mass may result in an underestimate of CRF.^21^, ^24^ Of note, 86% of the patients enrolled in the study had obesity at baseline, 22% had class III obesity (BMI ≥ 40 kg/m^2^) and 100% had either overweight or obesity. VAT allows the quantification of the ability to sustain submaximal physical activity for prolonged periods, which approximates levels associated with activities of daily living in individuals with HF, who rarely perform higher intensity activities.^6^ At the 12‐week visit, many subjects had a reduction in peak RER, which further reduced the power of our study to detect changes in peak VO_2_. Of note, an assessment of peak VO_2_ adjusted per RER showed a significant improvement with canagliflozin. It is unclear as to why subjects in the canagliflozin group exercised to a lower peak RER post‐intervention. This may be related to the imbalance in RER at baseline, with higher values in the canagliflozin group, but it could be related to other factors such as improved cardiac function allowing the individuals to reach the same or higher effort level while maintaining a greater cardiac reserve. It is indeed noteworthy that although canagliflozin patients tended to have lower RER at follow‐up this did not negatively affect the peak VO_2_, as it is was not reduced from baseline. It is possible that in this cohort of HFrEF patients with obesity and deconditioning, a treatment that improves cardiac function may fall short in improving peak VO_2_ due to supervening secondary limitation to maximal aerobic capacity.^25^

On the other hand, the VE/VCO_2_ slope, an effort‐independent CPX variable, was not affected by either treatment. Of note, while peak VO_2_ was clearly reduced in the investigated population (64% ± 12% of predicted), offering room for significant improvements, the mean VE/VCO_2_ slope was only mildly elevated (33.3 ± 7), with 11 subjects (31%) presenting with a normal VE/VCO_2_ slope (<30), potentially minimizing the ability to detect significant changes with the intervention.

Canagliflozin was associated with greater LVEF at 12 weeks compared to baseline, while sitagliptin had neutral effects, however, the time_x_group analysis was not statistically significant. Importantly, prior studies have shown favourable changes in systolic function (ie, LVEF and echocardiography‐derived strains) in preclinical models of HF.^26^ In addition to LVEF, we observed a small reduction in DT with canagliflozin compared to sitagliptin, which may reflect an improvement in myocardial relaxation. Of note, other more robust measures of cardiac diastolic function and estimate of filling pressures, such as E/E′, were not improved by canagliflozin.

These effects appear to be independent of glycemic control, which did not differ between the two groups. We did not observe significant changes in biomarkers of myocardial wall stress and renal function, such as NTproBNP and GFR, respectively. The lack of changes may be related to the study being underpowered to detect significant changes in such variables and/or short duration of treatment.

No changes in blood pressure and fluid status were found. While this may appear surprising, the baseline blood pressure and fluid status were within normal ranges, likely due to the fact that patients were required to be on maximally tolerated optimal medical therapy and stable without evidence of fluid overload prior to enrolment. We cannot exclude the possibility, however, that in this population, the blood pressure lowering effects of SGLT2 inhibitors^27^ may not be as pronounced, due to concomitant therapies and perhaps require a larger sample size to detect a significant change.^5^ We did not report any significant dietary changes in terms of daily caloric and sodium intake. We finally reported the safety profile of canagliflozin compared to sitagliptin in patients with established and well characterized HFrEF. This observation is, however, limited by the small sample size, the limited number of adverse events in the two groups, and the short duration of follow‐up.

Canagliflozin and other SGLT2 inhibitors have been shown to reduce hospitalizations for HF and renal events^2^, ^3^, ^4^, ^5^ in patients with T2DM and recently also in patients without T2DM.^5^, ^28^ Canagliflozin was shown to reduce cardiovascular and renal events, including hospitalizations for HF, even in patients with chronic kidney disease and albuminuria,^29^ further supporting its role in patients with T2DM to reduce cardiovascular events. In small studies, canagliflozin has been associated with improved diastolic function in patients with T2DM,^30^ however, its role on CRF and cardiac function in patients with established HF has not been previously investigated. We have previously shown that empagliflozin was associated with reduced E/E′ in patients with HFrEF^9^ suggesting a reduction in filling pressures and improvements in peak VO_2_ when patients where concomitantly treated with loop diuretics. Empagliflozin has also been associated with improved peak VO_2_ in other non‐randomized studies in patients with established HF.^10^ The recent EMPERIAL‐Preserved and EMPERIAL Reduced trials,^31^ however, suggested no improvements in functional capacity measured with 6‐minute walk test distance with empagliflozin. Of note, the EMPERIAL trials did not investigate the effects of SGLT2 inhibitors on CRF, particularly on peak VO_2_, like we attempted to do in our trial. Peak VO_2_ is a more sensitive parameter of exercise capacity than 6‐minute walk test distance, which instead estimates functional capacity and does not provide insights on the determinants of reduced CRF.^6^ Due to early interruption of our study, however, we cannot make definite conclusion on the effects of SGLT2 inhibitors on peak VO_2_, while we hope that ongoing clinical trials, such as the EMPA‐TROPISM will provide a final answer, also in patients without T2DM.^32^

The mechanisms responsible for such effects are largely unknown. The hearts of patients with HF present with metabolic dysregulation resulting in reduced ability to utilize carbohydrates as an energetic substrate, with a resulting compensatory increased utilization of fatty acids and ketone bodies. By increasing fatty acid oxidation, ultimately increasing the production of ketone bodies, SGLT2 inhibitors, may increase the availability and utilization of these newly preferred energetic substrates in the setting of HF.

Sitagliptin was neutral on CRF, QoL, cardiac function and biomarkers, confirming its safety profile explored in a large cardiovascular outcome trial.^11^

The study is not without limitations. In addition to the early termination, there were some differences in the baseline characteristics of the patients, which could have potentially affected the overall results of the study. Moreover, the small sample size of the study does not allow to definitely confirm the lack of differences of the safety profile of the two agents in the investigated population.

## 5 CONCLUSION

Despite the early interruption in enrolment and the failure to show differences in the primary endpoints of peak VO_2_ and VE/VCO_2_, we report for the first time that in patients with T2DM and HFrEF canagliflozin was associated with improvements in several measures of CRF such as lean peak VO_2_, VAT and RER‐matched VO_2_, and an improvement in the MLHFQ score reflecting a reduced HF symptom burden compared to sitagliptin, despite no significant differences in glycemic and hemodynamic control.

### Central Illustration. Key Findings of the Study.


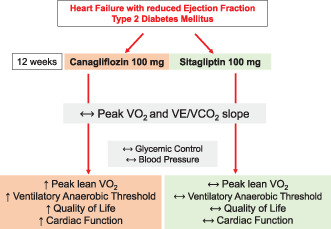


## CONFLICT OF INTEREST

Dr Abbate has served as consultant to Janssen. Dr Carbone is supported by a Career Development Award 19CDA34660318 from the American Heart Association. The other authors have nothing to disclose.

## Supporting information


**Supplemental Figure S1** Effects of treatments on blood pressure and body fluid. Canagliflozin nor sitagliptin were associated with significant changes in resting systolic blood pressure (BP) and diastolic BP (Panels A, B). Neither intervention affected body fluids, assessed with bioelectrical impedance analysis, including total body water (TBW) in litres (L) and percent (%) of body weight (Panels C, D), intracellular water (ICW) in L and % of TBW (Panels E, F), and extracellular water (ECW) in L and % of TBW (Panels G, H). All p values>0.05. Data are presented as mean ± SD.

## References

[1] Rawshani A , Franzen S , Sattar N , et al. Risk factors, mortality, and cardiovascular outcomes in patients with type 2 diabetes. N Engl J Med. 2018;379(7):633‐644. 30110583 10.1056/NEJMoa1800256

[2] Neal B , Perkovic V , Mahaffey KW , et al. Canagliflozin and cardiovascular and renal events in type 2 diabetes. N Engl J Med. 2017;377(7):644‐657. 28605608 10.1056/NEJMoa1611925

[3] Wiviott SD , Raz I , Bonaca MP , et al. Dapagliflozin and cardiovascular outcomes in type 2 diabetes. N Engl J Med. 2019;380(4):347‐357. 30415602 10.1056/NEJMoa1812389

[4] Zinman B , Wanner C , Lachin JM , et al. Empagliflozin, cardiovascular outcomes, and mortality in type 2 diabetes. N Engl J Med. 2015;373(22):2117‐2128. 26378978 10.1056/NEJMoa1504720

[5] McMurray JJV , Solomon SD , Inzucchi SE , et al. Dapagliflozin in patients with heart failure and reduced ejection fraction. N Engl J Med. 2019;381(21):1995‐2008. 31535829 10.1056/NEJMoa1911303

[6] Del Buono MG , Arena R , Borlaug BA , et al. Exercise intolerance in patients with heart failure: JACC state‐of‐the‐art review. J Am Coll Cardiol. 2019;73(17):2209‐2225. 31047010 10.1016/j.jacc.2019.01.072

[7] Greene SJ , Mentz RJ , Fiuzat M , et al. Reassessing the role of surrogate end points in drug development for heart failure. Circulation. 2018;138(10):1039‐1053. 30354535 10.1161/CIRCULATIONAHA.118.034668 PMC6205720

[8] Abbate A , Van Tassell BW , Canada JM , Dixon DL , Arena RA , Biondi‐Zoccai G . Pharmacologic and surgical interventions to improve functional capacity in heart failure. Heart Fail Clin. 2015;11(1):117‐124. 25432480 10.1016/j.hfc.2014.08.005

[9] Carbone S , Canada JM , Billingsley HE , et al. Effects of empagliflozin on cardiorespiratory fitness and significant interaction of loop diuretics. Diabetes Obes Metab. 2018;20(8):2014‐2018. 29603546 10.1111/dom.13309 PMC6043379

[10] Nunez J , Palau P , Dominguez E , et al. Early effects of empagliflozin on exercise tolerance in patients with heart failure: a pilot study. Clin Cardiol. 2018;41(4):476‐480. 29663436 10.1002/clc.22899 PMC6489772

[11] Green JB , Bethel MA , Armstrong PW , et al. Effect of sitagliptin on cardiovascular outcomes in type 2 diabetes. N Engl J Med. 2015;373(3):232‐242. 26052984 10.1056/NEJMoa1501352

[12] Association AD . Pharmacologic approaches to glycemic treatment: standards of medical care in diabetes‐2019. Diabetes Care. 2019;42(Suppl 1):S90‐s102. 30559235 10.2337/dc19-S009

[13] Davies MJ , D'Alessio DA , Fradkin J , et al. Management of hyperglycemia in type 2 diabetes, 2018. A Consensus Report by the American Diabetes Association (ADA) and the European Association for the Study of Diabetes (EASD). Diabetes Care. 2018;41(12):2669‐2701. 30291106 10.2337/dci18-0033 PMC6245208

[14] Canada JM , Fronk DT , Cei LF , et al. Usefulness of C‐reactive protein plasma levels to predict exercise intolerance in patients with chronic systolic heart failure. Am J Cardiol. 2016;117(1):116‐120. 26546248 10.1016/j.amjcard.2015.10.020

[15] Mitchell C , Rahko PS , Blauwet LA , et al. Guidelines for performing a comprehensive transthoracic echocardiographic examination in adults: recommendations from the American Society of Echocardiography. J Am Soc Echocardiogr. 2019;32(1):1‐64. 30282592 10.1016/j.echo.2018.06.004

[16] Trankle C , Canada JM , Buckley L , et al. Impaired myocardial relaxation with exercise determines peak aerobic exercise capacity in heart failure with preserved ejection fraction. ESC Heart Fail. 2017;4(3):351‐355. 28772034 10.1002/ehf2.12147 PMC5542717

[17] Garin O , Ferrer M , Pont A , et al. Disease‐specific health‐related quality of life questionnaires for heart failure: a systematic review with meta‐analyses. Qual Life Res. 2009;18(1):71‐85. 19052916 10.1007/s11136-008-9416-4

[18] World Health Organization . Waist circumference and waist‐hip ratio: report of a WHO expert consultation, Geneva, 8–11 December 2008: World Health Organization; 2011:39.

[19] Carbone S , Canada JM , Buckley LF , et al. Obesity contributes to exercise intolerance in heart failure with preserved ejection fraction. J Am Coll Cardiol. 2016;68(22):2487‐2488. 27908355 10.1016/j.jacc.2016.08.072 PMC5748881

[20] Carbone S , Canada JM , Buckley LF , et al. Dietary fat, sugar consumption, and cardiorespiratory fitness in patients with heart failure with preserved ejection fraction. JACC Basic Transl Sci. 2017;2(5):513‐525. 30062167 10.1016/j.jacbts.2017.06.009 PMC6058958

[21] Osman AF , Mehra MR , Lavie CJ , Nunez E , Milani RV . The incremental prognostic importance of body fat adjusted peak oxygen consumption in chronic heart failure. J Am Coll Cardiol. 2000;36(7):2126‐2131. 11127451 10.1016/s0735-1097(00)00985-2

[22] Van Tassell BW , Canada J , Carbone S , et al. Interleukin‐1 blockade in recently decompensated systolic heart failure: Results from REDHART (recently decompensated heart failure Anakinra response trial). Circ Heart Fail. 2017;10(11):e004373. https://doi.org/10.1161/CIRCHEARTFAILURE.117.004373 29141858 10.1161/CIRCHEARTFAILURE.117.004373 PMC5699505

[23] O'Connor CM , Whellan DJ , Lee KL , et al. Efficacy and safety of exercise training in patients with chronic heart failure: HF‐ACTION randomized controlled trial. JAMA. 2009;301(14):1439‐1450. 19351941 10.1001/jama.2009.454 PMC2916661

[24] Carbone S , Billingsley HE , Rodriguez‐Miguelez P , et al. Lean mass abnormalities in heart failure: the role of sarcopenia, sarcopenic obesity, and cachexia. Curr Probl Cardiol. 2019;100417. 10.1016/j.cpcardiol.2019.03.006 PMC11146283 31036371

[25] Van Tassell BW , Trankle CR , Canada JM , et al. IL‐1 blockade in patients with heart failure with preserved ejection fraction. Circ Heart Fail. 2018;11(8):e005036. 30354558 10.1161/CIRCHEARTFAILURE.118.005036 PMC6545106

[26] Santos‐Gallego CG , Requena‐Ibanez JA , San Antonio R , et al. Empagliflozin ameliorates adverse left ventricular remodeling in nondiabetic heart failure by enhancing myocardial energetics. J Am Coll Cardiol. 2019;73(15):1931‐1944. 30999996 10.1016/j.jacc.2019.01.056

[27] Baker WL , Buckley LF , Kelly MS , et al. Effects of sodium‐glucose cotransporter 2 inhibitors on 24‐hour ambulatory blood pressure: a systematic review and meta‐analysis. J Am Heart Assoc. 2017;6(5):e005686‐e. 28522675 10.1161/JAHA.117.005686 PMC5524106

[28] Zhou Y , Geng Z , Wang X , Huang Y , Shen L , Wang Y . Meta‐analysis on the efficacy and safety of SGLT2 inhibitors and incretin based agents combination therapy vs. SGLT2i alone or add‐on to metformin in type 2 diabetes. Diabetes Metab Res Rev. 2020;36(2):e3223. 31642583 10.1002/dmrr.3223

[29] Perkovic V , Jardine MJ , Neal B , et al. Canagliflozin and renal outcomes in type 2 diabetes and nephropathy. N Engl J Med. 2019;380:2295‐2306. 30990260 10.1056/NEJMoa1811744

[30] Matsutani D , Sakamoto M , Kayama Y , Takeda N , Horiuchi R , Utsunomiya K . Effect of canagliflozin on left ventricular diastolic function in patients with type 2 diabetes. Cardiovasc Diabetol. 2018;17(1):73. 29788955 10.1186/s12933-018-0717-9 PMC5963148

[31] Abraham WT , Ponikowski P , Brueckmann M , et al. Rationale and design of the EMPERIAL‐preserved and EMPERIAL‐reduced trials of empagliflozin in patients with chronic heart failure. Eur J Heart Fail. 2019;21(7):932‐942. 31218819 10.1002/ejhf.1486 PMC6774309

[32] Santos‐Gallego CG , Garcia‐Ropero A , Mancini D , et al. Rationale and design of the EMPA‐TROPISM trial (ATRU‐4): are the "cardiac benefits" of empagliflozin independent of its hypoglycemic activity? Cardiovasc Drugs Ther. 2019;33(1):87‐95. 30675708 10.1007/s10557-018-06850-0

