# Supplementary material for: The effects of canagliflozin compared to sitagliptin on cardiorespiratory fitness in type 2 diabetes mellitus and heart failure with reduced ejection fraction: The CANA‐HF study
Source: Diabetes Metab Res Rev. 2020 Jun 15;36(8):e3335. doi: 10.1002/dmrr.3335 (PMC7685099; doi:10.1002/dmrr.3335)
Supplement: Supplementary file 1 — Supplemental Figure S1 Effects of treatments on blood pressure and body fluid. Canagliflozin nor sitagliptin were associated with significant changes in resting systolic blood pressure (BP) and diastolic BP (Panels A, B). Neither intervention affected body fluids, assessed with bioelectrical impedance analysis, including total body water (TBW) in litres (L) and percent (%) of body weight (Panels C, D), intracellular water (ICW) in L and % of TBW (Panels E, F), and extracellular water (ECW) in L and % of TBW (Panels G, H). All p values>0.05. Data are presented as mean ± SD. [file DMRR-36-e3335-s001.docx]

**SUPPLEMENTAL MATERIAL**

**INCLUSION AND EXCLUSION CRITERIA**

**Inclusion criteria**

1) symptomatic stable HF (New York Heart Association [NYHA] class II-III) with a LVEF ≤40% measured within 6 months of enrollment and with no changes in cardiac medications within past 3 months);

2) peak exercise respiratory exchange ratio (RER)>1.00 at baseline CPX (reflecting a sufficient aerobic effort);

3) reduced peak aerobic exercise capacity (peak oxygen consumption [VO_2_]) <80% of the predicted value by age/sex;

4) T2DM (glycated hemoglobin [HbA1c] between 7.0% and 10.0% if on insulin, or between 6.5% and 10.0% if not on insulin;

5) willing and able to comply with the study protocol;

6) ≥18 years of age.

**Exclusion criteria**

1) Type 1 diabetes mellitus;

2) T2DM with episodes of severe hypoglycemia defined as glycemia <50 mg/dl by history, hypoglycemia unawareness by history, frequent changes in glucose-lowering agents in the past 3 months, or with prior episode of diabetic ketoacidosis;

3) open label treatment with SGLT2 inhibitors (within the past 3 months);

4) current treatment or within the past 3 months with thiazolidinediones;

5) participation in a structured exercise or weight loss program within the past 6 months;

6) chronic renal disease defined as an estimated glomerular filtration rate (GFR) <50 mL•min^-1^/1.73m^2^;

7) uncontrolled thyroid dysfunction (TSH<0.4 or >4.5 mcIU/mL);

8) pregnancy or of child-bearing potential or lactating;

9) active or recent (within 2 weeks) genital/urinary infection;

10) concomitant conditions or treatment which would affect completion or interpretation of the study including physical inability to walk or run on a treadmill such as decompensated HF (presence of edema, NYHA class IV), significant ischemic heart disease, angina, arterial hypotension, orthostatic arterial hypotension, uncontrolled arterial hypertension, atrial fibrillation with rapid ventricular response, severe valvular heart disease, severe chronic obstructive or restrictive pulmonary disease, moderate-severe anemia, diabetic neuropathy or myopathy;

11) abnormal blood pressure or heart rate response, angina or ECG changes (ischemia or arrhythmias) occurring during baseline CPX;

12) current or recent (within 2 weeks) use of oral corticosteroids;

13) inability to give informed consent.

**Supplemental Figure 1**. **Effects of treatments on blood pressure and body fluid.** Canagliflozin nor sitagliptin were associated with significant changes in resting systolic blood pressure (BP) and diastolic BP (**Panels A, B**). Neither intervention affected body fluids, assessed with bioelectrical impedance analysis, including total body water (TBW) in liters (L) and percent (%) of body weight (**Panels C, D**), intracellular water (ICW) in L and % of TBW (**Panels E, F**), and extracellular water (ECW) in L and % of TBW (**Panels G, H**). All p values>0.05. Data are presented as mean±standard deviation.
